# Medical and Insurance Problems

**Published:** 1908-11-21

**Authors:** 


### Medical and Insurance Problems.

The paper recently read at the opening meeting of the Life Assurance Medical Officers' Association by Professor Thomas Oliver on "Some Medical and Insurance Problems Arising out of Recent Industrial Legislation" contained much that is of interest to medical men. Several of the points touched upon by Professor Oliver are particularly interesting when read side by side with Dr. Seymour Taylor's lecture on "Malingering," which was published in The Hospital of October 17 last, and with Mr. Clinton Dent's address before the Medical Society on "The After-results of Injuries," which was considered in a leading article in the same issue. Professor Oliver dealt first with the advantages which injured workmen now possess through the operation of the latest Workmen's Compensation Act, for this has, as he says, placed them when in such circumstances beyond the reach of actual poverty. It is somewhat unsatisfactory to learn from him that in Germany, that pioneer country of industrial legislation, where insurance has been compulsory since 1883, experience shows that the burden imposed upon charity has not, as was expected, been lightened. In Germany the working classes have not improved in thrift, and we can scarcely hope that a similar cause acting in this country will produce a happier economic effect. In this connection the paper on "Social Insurances" read by Sir Edward Brabrook before the Royal Statistical Society on November 17 deserves especial notice.

Dr. Seymour Taylor stated from the results of his own experience, that since the passing of the Workmen's Compensation Acts of 1897 and 1906 the number of notified accidents and of claims for compensation has increased to a notable extent; and this is amply confirmed by Professor Oliver, who has made a special study of the available statistics. The former authority also stated that, in his opinion, owing to the effect of recent legislation, malingering is undoubtedly on the increase; and here again he receives corroboration in the paper under notice. It must be added, however, that Professor Oliver suggests that the increase of industrial accidents may possibly be due, in some degree, to carelessness in the supervision of work and machinery by employers, who, having insured against accidents, throw the whole burden of responsibility and risk upon the insurance offices. He speaks also of that sense of disability for work, more or less imagined, which so frequently develops after accidents, and which is so quickly dispelled by a settlement of the claim one way or the other. The working classes of both sexes, as he very justly points out, are increasingly subject to the widespread neurasthenia of modern days; and this factor has to be borne in mind whenever such questions are under consideration. In connection with this "disability to work" the interesting remarks by Mr. Clinton Dent on the mental and psychical after-effects of injuries in the police force will be called to mind. There can be little doubt that under certain circumstances a very slight objective injury may lead to real permanent disablement quite out of proportion to the physical cause.

As time goes on we shall learn more of the working of the recent Act and of its influence upon the health, safety, and morals of those whom it was principally designed to benefit. At present it is impossible to summon up much enthusiasm about it, either from the moral or the economical point of view. The working classes have certainly gained in the matter of provision during sickness, but beyond that the best that can be said of the Act is that up to now it has not led to that increase in the amount of litigation which was expected of it. The whole subject is of considerable importance to medical men of every standing, for in some form or another the question of compensation for injuries is constantly arising in practice, whether it be that of the consultant, of the general practitioner or of the resident medical officer.

